# Evaluation of Alcohol Preference and Drinking in msP Rats Bearing a *Crhr1* Promoter Polymorphism

**DOI:** 10.3389/fpsyt.2018.00028

**Published:** 2018-02-15

**Authors:** Marian L. Logrip, John R. Walker, Lydia O. Ayanwuyi, Valentina Sabino, Roberto Ciccocioppo, George F. Koob, Eric P. Zorrilla

**Affiliations:** ^1^Committee on the Neurobiology of Addictive Disorders, The Scripps Research Institute, La Jolla, CA, United States; ^2^Department of Psychology, Indiana University – Purdue University Indianapolis, Indianapolis, IN, United States; ^3^Genomics Institute of the Novartis Research Foundation, San Diego, CA, United States; ^4^Pharmacology Unit, School of Pharmacy, University of Camerino, Camerino, Italy; ^5^Laboratory of Addictive Disorders, Department of Pharmacology and Experimental Therapeutics, Boston University School of Medicine, Boston, MA, United States; ^6^Neurobiology of Addiction Section, Integrative Neuroscience Research Branch, National Institute on Drug Abuse, Baltimore, MD, United States; ^7^Department of Neuroscience, The Scripps Research Institute, La Jolla, CA, United States

**Keywords:** alcohol, ethanol, corticotropin-releasing factor, corticotropin-releasing hormone, promoter, single-nucleotide polymorphism, genetic vulnerability, selective breeding

## Abstract

Alcoholism is a pervasive societal problem, yet available pharmacotherapies fail to treat most sufferers. The type 1 corticotropin-releasing factor (CRF_1_) receptor has received much attention for its putative role in the progression to alcohol dependence, although at present its success in clinical trials has been limited. Two single-nucleotide polymorphisms in the rat *Crhr1* promoter have been identified in the Marchigian substrain of Sardinian alcohol-preferring (msP) rats. Unlike other Wistar-derived alcohol-preferring lines, nondependent msP rats reduce their alcohol self-administration in response to CRF_1_ antagonists and show increased brain CRF_1_ expression. The current study tested the hypotheses that the A alleles in the *Crhr1* promoter polymorphisms are: (1) unique to msP (vs. CRF_1_ antagonist-insensitive) alcohol-preferring lines and (2) associate with greater alcohol preference or intake. Two related polymorphisms were observed in which both loci on a given chromosome were either mutant variant (A) or wild-type (G) alleles within the distal *Crhr1* promoter of 17/25 msP rats (68%), as compared to 0/23 Indiana P rats, 0/20 Sardinian alcohol-preferring rats bred at Scripps (Scr:sP) and 0/21 outbred Wistar rats. Alcohol consumption in msP rats did not differ according to the presence of *Crhr1* A alleles, but greater alcohol preference (98%) was observed in A allele homozygous msP rats (AA) compared to msP rats with wild-type (GG, 91%) or heterozygous (GA, 91%) genotypes. The greater alcohol preference reflected decreased water intake, accompanied by reduced total calories consumed by AA rats. The data show that msP rats differentially possess mutant A variant alleles in the polymorphic promoter region of the *Crhr1* gene that may differentially regulate consumption.

## Introduction

Alcohol abuse and dependence affect about 8% of the population worldwide (1), generating significant societal costs (2). Despite this major public health burden, available pharmacotherapies for alcohol use disorders remain inadequate. Toward developing new therapeutic targets, rodent lines have been selectively bred for differential propensity to consume alcohol. The characterization of genetic differences in these lines may identify new molecular targets for medications development.

One target implicated in the progression and persistence of alcohol dependence is corticotropin-releasing factor (CRF), a peptide upregulated and released in the extended amygdala during alcohol withdrawal (3–6). Alcohol dependence confers sensitivity to CRF_1_ antagonists to reduce alcohol self-administration (7–9), similarly to binge-like alcohol consumption observed in intermittent access (10–13) and “Drinking in the Dark” (DID) paradigms (14–17). Blockade of CRF_1_ does not typically alter alcohol intake in nondependent rodents consuming alcohol in non-binge-like patterns (7, 13–15). Sensitization of the CRF system has been hypothesized to mediate the transition to dependence, whereby negative reinforcement (“self-medication”) putatively drives high alcohol intake in situations of CRF system hyperactivation (18–20). While CRF_1_ antagonists have so far proved largely unsuccessful to treat alcohol use disorders in human clinical trials (21, 22), this has been theorized to result from individual variation in clinical trial subjects’ drinking motivation, such that only a subset of the population, such as those engaging in stress-induced drinking, might respond to CRF_1_ antagonist treatment [reviewed in Ref. (23)]. Thus understanding the role of CRF in promoting escalated alcohol intake in rodent models remains important. The presence of heightened CRF system activity in rat lines bred for high alcohol preference may recapitulate the behaviors that result from extensive alcohol exposure, and consequent recruitment of CRF circuitry, that are seen in environmental rodent models of alcohol dependence.

Several alcohol-preferring rat lines have been selectively bred from Wistar stock, including the Indiana alcohol-preferring [P (24)] and Sardinian alcohol-preferring [sP (25)] lines, as well as substrains derived from the sP line, including Marchigian sP [msP (26, 27)] and Scripps sP [Scr:sP (9, 28, 29)] rats. Different genetic factors may underlie each line’s excessive alcohol drinking. Studies suggest that the lines are not uniform in their CRF/CRF_1_ systems. For example, Scr:sP rats show elevated amygdala dialysate CRF levels relative to Sardinian non-preferring rats (30), and msP rats exhibit increased CRF_1_ expression in the extended amygdala relative to Wistar rats (31, 32). In contrast, P rats show reduced CRF expression, as compared to non-preferring rats, in the amygdala and cortex (33). Furthermore, despite similarly high alcohol intake, only msP rats (31, 34, 35), and not Scr:sP (9) or P (36) rats, reduce their basal levels of operant alcohol self-administration in response to CRF_1_ antagonists, a trait that may result from the msP rats’ elevated basal anxiety (32, 35).

Previously, two single-nucleotide polymorphisms (SNPs) were discovered in the distal promoter region of the CRF_1_ gene (*Crhr1*), with 85% of msP rats vs. 0% of Wistar controls possessing an A allele at both polymorphic positions (A allele) vs. the typical G allele (31). The variant A allele was hypothesized to contribute to the increased CRF_1_ expression and CRF_1_ antagonist sensitivity of msP rats. However, it remains unknown whether the mutant variant A allele is unique to msP rats, relative to other alcohol-preferring lines (Scr:sP, P). Here, we tested the hypotheses that (1) the presence of the variant A allele in the *Crhr1* promoter region is restricted to the CRF_1_ antagonist-sensitive msP line and (2) the presence of two mutant (A) alleles is associated with elevated home cage alcohol intake or preference in the msP line. Our results demonstrate that the presence of the A allele is unique to the msP strain, and that homozygous expression of the A allele (AA) was not differentially associated with elevated baseline alcohol consumption in msP rats, although the AA genotype may associate with a slight increase in alcohol preference, albeit over very high preference levels in all msP rats.

## Materials and Methods

### Ethics Statement

These studies were carried out in accordance with the recommendations of the European Community Council Directive for the Care and Use of Laboratory Animals and the University of Camerino Internal Ethical Committee for Laboratory Animal Protection and Use (CEAPA), or in accordance with the NIH Guide for the Care and Use of Laboratory Animals and The Scripps Research Institute Institutional Animal Care and Use Committee (IACUC). The protocols were approved by the University of Camerino CEAPA and by the Scripps Research Institute IACUC.

### Animals

Subjects were adult males from two distinct sublines derived from the Sardinian preferring (sP) rats—the msP and Scr:sP rats. The breeding program of sP rats began in 1981 (University of Cagliari, Italy). Beginning with the 13th generation of sP rats, a subset of rats was provided to a separate program for selective breeding at the University of Camerino, Italy. After 20 generations of selective breeding for high alcohol preference, this distinct subline was renamed msP (27). The msP rats in the present study (*n* = 25 for the strain comparison and *n* = 79 for the home cage consumption study) were from the 58th–59th generations of selective breeding. A rederivation of the msP line, to generate sublines homozygous for the G allele (GG) and homozygous for the A allele (AA), was performed as described in Ref. (35). Rats from these sublines (*n* = 9 GG, *n* = 8 AA) were used for analysis of food intake when no alcohol was available. All behavioral studies, which involved only msP rats, were performed at the University of Camerino (Camerino, Italy). While these studies only include male subjects, it should be noted that sex differences in CRF_1_ signaling have been reported in the locus coeruleus (37) that might increase female responsiveness to conditions of altered CRF_1_ expression, although sex differences were not observed following germline *Crhr1* deletion (38).

In the 32nd generation of selective breeding of sP rats according to high alcohol preference at the University of Cagliari, a subset of sP rats were provided by Dr. G. L. Gessa to The Scripps Research Institute and maintained as a colony without further selective breeding. Rats from this colony were designated as Scr:sP (http://rgd.mcw.edu/rgdweb/report/strain/main.html?id=2302666). The Scr:sP subline, like the sP line from which they were derived (39), show high spontaneous home cage alcohol intake (5–7 g/kg daily) and preference (>80% vs. water) and display high levels of anxiety-like behavior (9, 40). The Scr:sP rats in the present study (*n* = 20) were from the 23rd–24th generations of intra-line breeding at the Committee on the Neurobiology of Addictive Disorders of The Scripps Research Institute (La Jolla, CA, USA).

For the strain comparison, adult male outbred Wistar rats (*n* = 21) obtained from Charles River (Raleigh, NC, USA) were used as controls. In addition, adult male Indiana P rats (41), generously provided by Dr. L. Lumeng (Indiana University School of Medicine, Indianapolis, IN, USA), also were subjects (*n* = 23) at The Scripps Research Institute. P rats have been bred at Indiana University School of Medicine since original establishment of the line by selective breeding from a Wistar rat colony at the Walter Reed Army Hospital (the Walter Reed Wistar rat) (42). P rats demonstrate high alcohol intake in the home cage (>5 g/kg/day) (43), yielding pharmacologically relevant blood alcohol levels (44).

Rats were housed under a 12-h reversed light cycle under constant temperature (20–22°C) and humidity (45–60%). Rats were housed three per cage in standard, wire-topped plastic cages containing sawdust bedding, with food (Camerino: 4RF18, Mucedola, Settimo Milanese, Italy; Scripps: LM-485 Diet 7012, Harlan, Madison, WI, USA) and water available *ad libitum*. Rats for behavioral studies received environmental enrichment prior to experimentation, at which time rats were singly housed to allow for recording of drinking and food consumption, as detailed below. At the conclusion of experiments, rats were euthanized by carbon dioxide inhalation.

### Sequencing of the *Crhr1* Promoter

**Figure 1:**
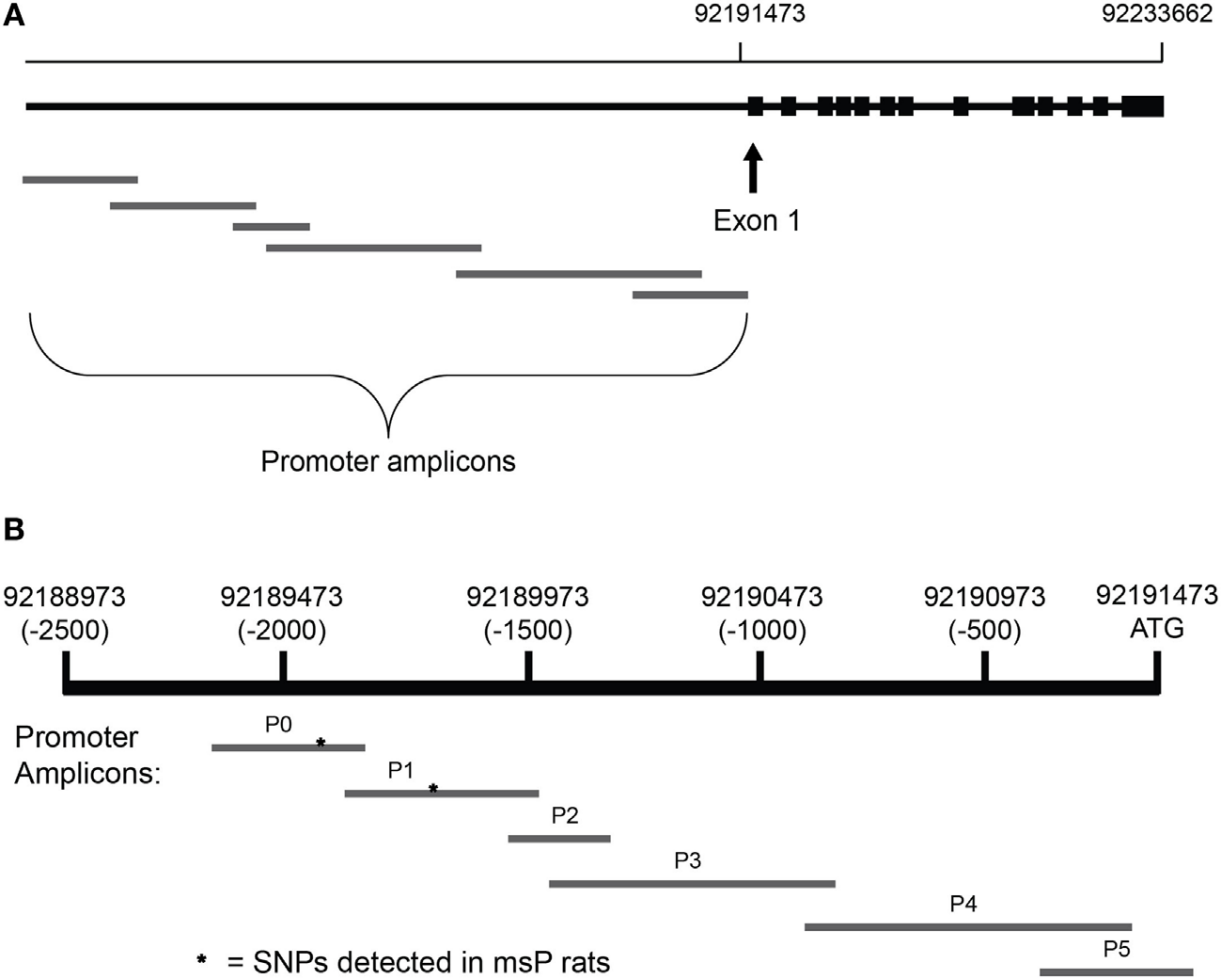
Alignment of amplicons used for *Crhr1* promoter sequencing. Primer pairs were designed using the *Crhr1* promoter sequence obtained from the University of California, Santa Cruz genome browser to generate six overlapping amplicons for complete coverage of the promoter region, based in part on previously published sequences (31). Chromosomal alignment of the overlapping *Crhr1* promoter amplicons is shown relative to **(A)** the *Crhr1* gene sequence and **(B)** the *Crhr1* proximal promoter region (based on UCSC Genome Browser, rat assembly July 2014). While consistent with the relative lengths of amplicons produced, the visual representation is not drawn to scale.

*Crhr1* promoters were sequenced in all subjects. For the strain comparison, 6 primer pairs were designed to sequence the ~2,300 bases upstream of the *Crhr1* start codon (Figure 1A) and obtained from Integrated DNA Technologies (Coralville, IA, USA). The target amplicons, named P0-P5, were selected to generate an accurate, complete sequence of the proximal promoter region using slight overlap between all adjacent amplicons (Figure 1B). Primer pairs P0 and P1 were designed to overlap with the promoter sequence in which the msP *Crhr1* polymorphisms were reported previously (31), with each amplicon containing one of the two known SNPs, to ensure successful sequencing. Primer sequences are provided in Table 1. Tail DNA was extracted and polymerase chain reaction (PCR) was carried out with all samples (~30 ng DNA/well) run on a single 96-well plate for each primer pair with seven wells reserved as no template controls. Sequencing reactions were run in both the forward and reverse directions. ABI trace files for all high quality sequencing results (defined as Phred scores > 20 for at least 75% of the sequence) were analyzed using Mutation Surveyor. Only mutations identified in analysis of both the forward and reverse direction sequencing reactions were considered to be real mutations. This approach for complete sequencing of the 2,300 bp upstream of the *Crhr1* start codon was utilized for the strain comparison study.

**Table 1:** *Crhr1* promoter primer sequences.

Amplicon	Forward primer	Reverse primer
P0	TGAAATCTGCTGCTTACTGAGCCC	TGGGCAAGGAATGCGTACCTCTTA
P1	AGCACTTTCCCTCCAACAACCCTA	ACTCTGTTCTCAGCACACTGGACA
P2	ATCGCATGACCTACAGCAACTCCA	TCTTGAGTACCCAGAAGCACCGAA
P3	TGGATCTTGTCCAGTGTGCTGAGA	AACTAAGCGTCTGTCTGTTTGGTC
P4	AGCTTCAGTGTCTCAGCACATCCT	AAGTCTGGTAGCCTTCTCCCGA
P5	AGAGGAGGGAGAAAGAGGAGGG	GCTTCAGAGATCCAGGTAGAGGACAT

Once it was clear which *Crhr1* promoter nucleotide positions were likely to be altered in msP rats, a more targeted genotyping approach was taken for the rats involved in the behavioral studies. Because only 2 polymorphisms were observed across all rats in the strain comparison study, PCR primers were designed to target only the expected polymorphism sites, encompassing positions −2,092 and −1,835 bp relative to ATG start of *Crhr1*. Primer extension assays were performed using the Sequenom iPlex Gold reagent kit (San Diego, CA, USA), and extension products analyzed by mass spectrometry using a Sequenom Mass Array system. Base calls scored as “low probability” by the Sequenom software were not used in the final analysis.

### Home Cage Food and Alcohol Intake

To explore further the functional significance of the *Crhr1* promoter genotype within the msP line, behavioral studies were performed in male msP rats (*n* = 79, weight range during experimentation 333–536 g, run in two cohorts of 32 and 47 rats, respectively). Rats were single-housed prior to the first alcohol access day to quantify home cage intake. To ascertain the relation of *Crhr1* promoter genotype to home cage alcohol intake, msP rats were provided *ad libitum* concurrent, continuous access to 10% alcohol (0.56 kcal/ml), water and food pellets (Mucedola 4RF18, 2.6 kcal/g) until a stable baseline alcohol intake (5–7 g/kg/day) was achieved (maximum 15% daily variation, reached in 3 weeks). Alcohol was prepared as a 10% (*v/v*) solution in tap water, and intake of both solutions and food was measured daily at 2, 4, 8, 12, and 24 h after the onset of the dark cycle. As no time-dependent differences were observed, data are presented as 7-day means of 24-h intake. All consumption measures are normalized to body weight to account for size differences in both caloric requirement and pharmacological efficacy. To assess differences in focus on alcohol as a fluid or caloric source, data are additionally presented as percent alcohol preference [100 × 10% (*v/v*) alcohol intake/total fluid intake] and as percent calories derived from alcohol (100 × alcohol intake in kcal/total kcal intake), per (17, 45). To further assess the impact of alcohol availability on chow intake, an additional group of rats from a rederivation of the msP line to separately breed AA and GG homozygous rats (35) was provided with *ad libitum* access to food pellets (Mucedola 4RF18, 2.6 kcal/g) and water in the absence of alcohol. Data are presented as 3-day means of 24-h food intake.

### Data Analysis

Strain differences in allele frequency were determined by Pearson’s chi square analysis. Alcohol intake, water intake and alcohol preference data were not normally distributed, possibly due to floor and ceiling effects, respectively, and thus were analyzed by ANOVA on ranks rather than ANOVA on raw values, with *post hoc* comparisons performed using Dunn’s method. Body weight, food intake and caloric intake data were found to conform to normal distributions, and thus were analyzed by one-way ANOVA, with *post hoc* comparisons *via* Tukey’s test. SigmaPlot 13.0 and Systat 13 (Chicago, IL, USA) software were used to perform the statistical analyses.

## Results

### *Crhr1* Promoter SNP Prevalence in Wistar-Derived Alcohol-Preferring Rat Strains

Previously, two G-to-A base pair substitutions were reported 2,097 and 1,836 bases upstream from the start codon in the *Crhr1* promoter region of most (85%) msP rats (31). Here, we sought to determine whether the A alleles are a common modification within Wistar-derived alcohol-preferring rat strains or are a distinguishing feature of msP rats that may account for their unique sensitivity to CRF_1_ antagonists under non-dependent conditions (9, 31, 36). Two statistically related polymorphisms were observed in the *Crhr1* promoter in which both loci were either mutant variant (A) or wild-type (G) alleles. One SNP, from primer pair P0, was at position −2,092 relative to the ATG start site for *Crhr1* on rat chromosome 10 (UCSC Genome Browser rat assembly November 2004: position 93,310,313 vs. ATG start at 93,312,405; rat assembly March 2012: position 91,951,378 vs. ATG start at 91,953,470; and rat assembly July 2014: position 92,189,381 vs. ATG start at 92,191,473). The second SNP, from primer pair P1, was at position −1,835 (UCSC Genome Browser rat assembly November 2004 position 93,310,570; rat assembly March 2012, position 91,951,635; rat assembly July 2014, position 92,189,638). The discrepancy in these SNP positions relative to the *Crhr1* start codon, previously reported at 2,097 and 1,836 bases upstream of transcription initiation (31), may be due to updates in the genome sequence since the initial publication of the SNPs but is consistent across the 2004, 2012, and 2014 UCSC Genome Browser assemblies. No additional high-frequency polymorphisms, defined as greater than 50% prevalence in any one or more of the strains studied, were identified in the promoter region. As shown in Table 2, the joint mutant variant A alleles at the polymorphic *Crhr1* promoter loci were common in (68%) and exclusive to the msP line (χ^2^ = 53.80, *p* < 0.001). *Post hoc* comparisons demonstrated elevated prevalence of the polymorphisms in the msP strain as compared to all other strains tested (all χ^2^’s > 19.06, all *p*’s < 0.001). Only one P rat carried the −2,092 G-to-A SNP, but this was without the −1,836 SNP that invariably accompanied it in msP rats. Thus, the *Crhr1* promoter A allele, with A at both polymorphic positions, was unique to the msP line.

**Table 2:** Prevalence of *Crhr1* promoter polymorphisms in normal and alcohol-preferring rat strains.

Strain	Subjects	Double SNP expressing	Prevalence (%)
Wistar	21	0	0.0
Indiana P	23	0^a^	0.0
Scr:sP	20	0	0.0
msP	25	17	68.0***

***p < 0.001 vs. all other groups.

*^a^One Indiana P rat (4.3%) displayed one but not both SNPs*.

### Relation of the *Crhr1* Promoter SNPs to Alcohol Intake and Preference

**Figure 2:**
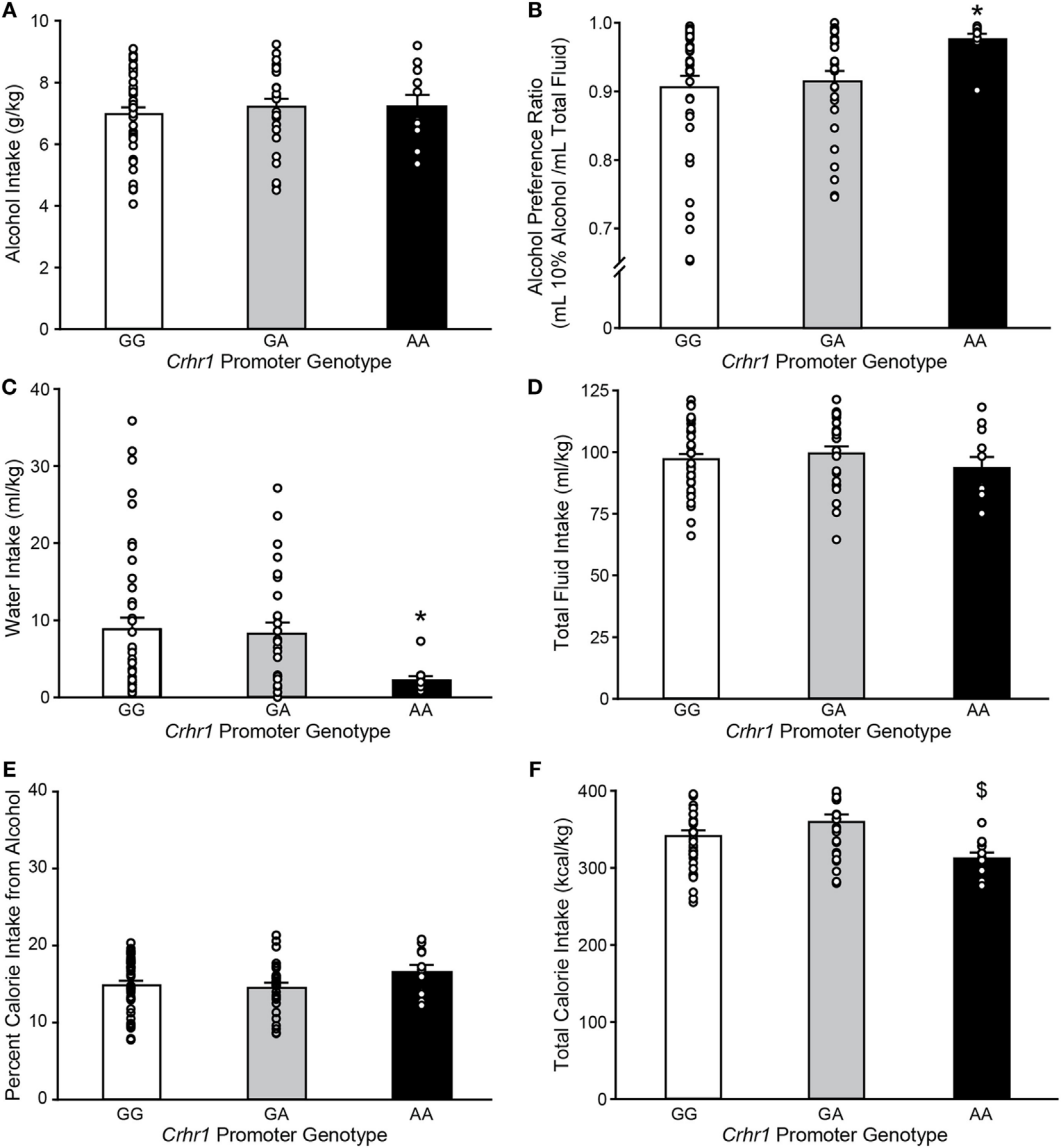
Marchigian substrain of Sardinian alcohol-preferring (msP) rats carrying the *Crhr1* promoter polymorphisms on both alleles show altered alcohol preference, water intake and calories consumed. Home cage fluid intake and preference were determined for msP rats with known *Crhr1* promoter genotypes consuming 10% alcohol under a two-bottle choice paradigm. Genotype-specific effects were assessed for **(A)** alcohol intake, **(B)** alcohol preference [(alcohol consumed)/(total fluid intake)], **(C)** water intake, **(D)** total fluid intake, **(E)** percent calories obtained from alcohol [(alcohol calories consumed)/(total caloric intake)], and **(F)** total calorie intake. Data are expressed as mean ± SEM daily measurements averaged across 7 days of stable intake. Overlaid on histograms are individual subjects’ data points to demonstrate the variability among subjects in each group. *p < 0.05 vs. GG and GA, ^$^*p* < 0.05 vs. GA. *n* = 11 AA, 27 GA, 41 GG.

As the recently reported analysis of brain region-specific *Crhr1* expression was performed in rederived lines of GG and AA msP rats (32), it was of interest to determine whether the presence of G vs. A alleles had been associated with differences in alcohol consumption in the parent msP strain, in which elevated *Crhr1* expression was more widespread than observed in the rederived lines (31). msP rats from the parent strain were provided continuous two-bottle choice access to 10% alcohol vs. water in the home cage, and *Crhr1* genotype determined after collection of all behavioral data. The A allele was present in approximately half of the msP rats in the drinking study (48.1%), with the AA genotype representing a minority of msP rats (11/79 = 13.9%) as compared to GA (27/79 = 34.2%) and GG (41/79 = 51.9%). As shown in Figure 2, weight-normalized alcohol consumption did not differ according to *Crhr1* promoter allele (Figure 2A; one-way ANOVA on Ranks, *H* = 0.64, *p* = 0.73), similar to published data for rederived GG vs. AA msP sublines (35). However, a significant elevation in alcohol preference was displayed by homozygous AA msP rats, relative to rats with only one A allele (GA) and rats homogyzous for the G allele (Figure 2B; one-way ANOVA on ranks, *H* = 8.11, *p* < 0.05). Preference differences reflected that AA rats consumed similar quantities of alcohol, but drank strikingly less water (Figure 2C; one-way ANOVA on ranks, *H* = 7.41, *p* < 0.05), without significant difference in total fluid intake (Figure 2D; one-way ANOVA, *F*_2,76_ = 0.71, *p* = 0.49).

AA rats also showed nonsignificant trends to eat less food (one-way ANOVA, *F*_2,76_ = 2.65, *p* = 0.08), yet weigh more (*F*_2,76_ = 2.75, *p* = 0.07), as shown in Table 3. Because alcohol provides a source of calories, the increased preference and reduced food intake suggested a possible shift toward obtaining calories from alcohol. To investigate this possibility, the percent of total calories obtained from alcohol was calculated. *Crhr1* promoter genotype did not significantly alter the use of alcohol as a calorie source (Figure 2E, one-way ANOVA, *F*_2,76_ = 1.39, *p* = 0.26), although genotype did significantly impact total caloric intake (Figure 2F, one-way ANOVA, *F*_2,76_ = 4.09, *p* < 0.05), with AA rats consuming significantly fewer calories than GA, but not GG, rats. These data suggest a role for *Crhr1* promoter genotype in regulating caloric intake, but the possibility remained that alcohol availability might alter food intake. To assess *Crhr1* promoter genotype effects on food intake under baseline conditions, when alcohol was not available, food consumption was measured in AA and GG rats from the rederived msP lines (35) over a 3-day period. As shown in the bottom half of Table 3, AA and GG rats of the rederived lines consumed similar quantities of food (one-way ANOVA, *F*_1,15_ = 0.45, *p* = 0.51) and did not differ in body weight (one-way ANOVA, *F*_1,15_ = 0.04, *p* = 0.84).

**Table 3:** Food intake and body weight of msP rats with home cage two-bottle choice access to 10% alcohol.

Promoter genotype	Food (kcal)	Weight (g)
**10% alcohol available concurrently**		
AA	117.8 ± 2.4	452.7 ± 6.5
GA	128.4 ± 2.7	421.3 ± 6.8
GG	124.8 ± 2.1	433.4 ± 6.6
**No alcohol available**		
AA	57.3 ± 1.7	415.4 ± 8.1
GG	54.3 ± 1.8	417.7 ± 8.0

*Food intake and body weight expressed as mean ± SEM*.

## Discussion

The present data demonstrate that the A allele polymorphisms in the *Crhr1* promoter region are specific to msP rats and likely play a minor role in promoting alcohol drinking under unstressed conditions. The two previously described (31) G-to-A SNPs were confirmed in the *Crhr1* promoter of msP rats, but were not found to be common markers of high alcohol intake, as they were not observed in other alcohol-preferring strains selectively bred from Wistar stock (P, Scr:sP). Rather, the presence of A nucleotides at both polymorphic loci was nearly exclusively restricted to the msP line, with only one rat (4%) from the Indiana P line presenting an A nucleotide at one polymorphic locus, but not both, on a single allele. Within the msP line, the mutant A variant allele was associated with decreased water intake, lower total caloric intake, and elevated alcohol preference, although preference differences may have minimal physiological impact as all preference scores were extremely high regardless of genotype, and alcohol consumption did not differ. Recently, the msP line was rederived to generate parallel lines homozygous for A alleles (AA) or wild-type G alleles (GG) (35). In the rederived msP AA rats, elevated alcohol preference was visible but did not reach significance, except under conditions of heightened stress (35). Similarly, we observed reduced caloric intake in AA rats from the parent strain that was not seen in rederived msP AA rats, when assessed in the absence of alcohol availability. These data suggest that the alcohol preference and caloric intake phenotypes may either require additional interacting gene variants possessed by the parent, but not rederived, line, be determined (partly) independent of the *Crhr1* promoter polymorphisms, or be triggered by differential life experiences, like stressors, that could have differentially affected a specific cohort and would be hypothesized to increase *Crhr1* expression to a greater degree in msP AA rats (35). In the rederived rats, both GG and AA rats displayed elevated amygdala *Crhr1* mRNA levels relative to Wistar controls, whereas *Crhr1* levels in the bed nucleus of the stria terminalis (BNST) were higher in AA vs. GG rats (32). Collectively, the data demonstrate that the mutant A variant allele at the polymorphic *Crhr1* promoter loci is restricted to msP rats and may be related to elevated alcohol preference, but it is not sufficient to promote greater alcohol drinking or amygdala CRF_1_ expression observed in the msP rat line under unstressed conditions (31). However, the polymorphisms have been associated with increased stress sensitivity (32, 46) and heightened response to CRF_1_ antagonism (31, 34, 35) in both the parent and rederived lines, suggesting that both the drinking phenotype and the CRF_1_ antagonist sensitivity may relate to the significantly elevated anxiety-like behavior generally displayed by msP rats (32, 46). While operant self-administration was elevated by the pharmacological stressor yohimbine in both GG and AA rats, the contribution of the gene variants to drinking under stress remains to be determined.

### *Crhr1* Polymorphisms and the Regulation of CRF_1_ Expression

Hansson et al. originally reported 2 G-to-A substitutions in the promoter region of *Crhr1* of msP rats on one or both gene loci in a majority (85%) of msP rats (31). The present study found lower frequency of the A nucleotides, occurring in 68% of msP rats in the strain survey and 48% of msP rats in the behavior cohort. These frequency differences may result from random selection of rats from the population for the various experiments, as *Crhr1* promoter genotype was not determined until the conclusion of experiments, or from genetic drift in the frequency of the AA loci in the msP parent line over time. The rederived parallel AA and GG msP lines now allow for direct assessment of the behavioral impact of the promoter polymorphism (35), particularly helping to delineate the phenotypes observed in the parent msP line that may have resulted from genes other than *Crhr1* cosegregating with AA status.

In addition to the reduced frequency of the dual polymorphism in the current msP cohort, slight differences in the locations of both SNPs were identified in the genetic analysis, with one located 1,835 bp (rather than 1,836 bp) upstream of the receptor start codon, and the second SNP at 2,092 bp (rather than 2,097 bp) upstream. This discrepancy in position may reflect modification of the genome sequence since the original identification of the polymorphisms. Both loci lie well outside the proximal core promoter region (47), which could explain the apparent lack of involvement of the variant alleles in basal amygdala *Crhr1* expression (31, 32). However, in the rederived msP lines, *Crhr1* expression in the BNST was higher in AA than in GG or Wistar controls, suggesting that the polymorphism can affect *Crhr1* gene expression in a brain region-specific manner, despite the distance from the core promoter region. Distal DNA regions as far away as 100 kb can alter transcription *via* various mechanisms, resulting in enhancement, repression or insulation of transcription factor activity (47–50). Such effects can occur *via* looping of the intervening DNA sequence that apposes the distal upstream sequence to the proximal promoter (49, 51), as well as by regulation of DNA availability through methylation (50). Despite these possible mechanisms by which distal *Crhr1* promoter regions may regulate its expression, at present the data are largely lacking for genotype differences in basal *Crhr1* levels. Nonetheless, distal regions of the *Crhr1* promoter may provide a site for experience-based modulation of *Crhr1* transcription that would confer the differential sensitivity to CRF_1_ antagonism and stress-induced reinstatement observed in AA msP rats (35).

One mechanism by which stress may alter CRF_1_ function, *via* altered receptor expression, involves changes in distal promoter methylation status. The *Crhr1* promoter region lies within a CpG island, and behavioral experiences that alter anxiety-like behavior have been shown to increase methylation on one CpG site located 1,348 bases upstream of the *Crhr1* translation start in the amygdala (52). Recently, stress-related changes in gene methylation status and expression of another receptor (*Oprl1*) were shown in msP rats using a stress paradigm that increased msP rats’ alcohol consumption (53), indicating that stressors that exacerbate alcohol intake in msPs might accomplish this *via* altered methylation, and thereby expression, of specific gene targets. Since AA msP rats are anticipated to be more susceptible to stress-induced elevations in drinking based on yohimbine exacerbation of operant alcohol self-administration (35), stress-related increases in *Crhr1* promoter methylation might differentially impact *Crhr1* gene expression in AA vs. GG msP rats. Together these data highlight the possible involvement of distal regions of the *Crhr1* promoter as putative sites of stress-related regulation of CRF_1_ expression, and point to the identification of the molecular mechanisms by which the variant A alleles may confer increased sensitivity to stress and CRF_1_ modulation of alcohol self-administration as an important focus to uncover new therapeutic avenues for alcohol dependence.

### CRF_1_ Regulation of Alcohol Intake

Increased activation of the CRF system is evident in the progression to substance dependence, including for alcohol [reviewed in Ref. (54)]. Pharmacological and genetic studies specifically implicate activation of extended amygdala CRF_1_ receptors in mediating the excessive alcohol self-administration resulting from chronic intermittent exposure (7, 8) or access to alcohol sufficient to produce excessive (10) or binge-like (11, 15–17) drinking. Before CRF_1_ antagonists were found to reduce alcohol self-administration in nondependent msP rats (31), recruitment of the CRF system had been conceptualized, even within alcohol-preferring rat lines (9, 36), as a mechanism of transition to alcohol dependence that resulted from chronic intermittent alcohol exposure. Indeed, chronic intermittent exposure to alcohol in Wistar rats leads to alcohol dependence and increased alcohol intake in association with increased extracellular CRF levels during withdrawal (6), depletion of amygdala CRF tissue content (3, 55), elevated CRF_1_ expression in the basolateral and medial amygdala (56), and potentiation of GABA and glutamate release in the central amygdala (57, 58). msP rats also express elevated CRF_1_, even under alcohol-naïve conditions (31), and show elevated function of the GABA and glutamate systems in the central amygdala (46, 59, 60). Binge-like DID alcohol consumption in C57BL/6J mice also altered CRF levels and electrophysiological properties of the central amygdala (15), but in contrast to alcohol dependence and msP findings, CRF immunoreactivity increased, presynaptic GABA release was unchanged, and CRF lost the ability to enhance evoked GABAergic currents after DID. Thus, CRF_1_ antagonists’ ability to similarly reduce alcohol consumption in multiple models of heightened alcohol intake may arise from differential adaptation at the neuronal level based on alcohol access parameters utilized and stimuli producing heightened alcohol intake (7, 8, 11, 12, 16, 34, 61–63). The regulation of alcohol intake by CRF_1_ encompasses its activation by both CRF and urocortin-1, which has been shown to specifically regulate escalating, but not controlled, alcohol intake (45). How urocortin-1 impacts alcohol intake in preferring rat lines, including msP rats, and whether this might be a site of neuronal adaptation yielding high alcohol intake remain to be determined, although greater density of urocortin-1 fibers across multiple preferring, vs. non-preferring, rat lines (64) suggest likely contribution of urocortin-1 to the high drinking phenotype. Surprisingly, while the msP (27), P (41), and Scr:sP (9, 40, 65) strains of alcohol-preferring rats each display high levels of alcohol intake, only msP rats are sensitive to CRF_1_ antagonists in the absence of experimentally induced alcohol dependence (9, 27, 35, 36). The alcohol dependent-like neuronal activity profiles displayed in the central amygdala of msP rats (46, 59, 60), which may contribute to the rats’ increased anxiety-like phenotype (32, 46), are of particular interest for further examination as a basis for msP rats’ high alcohol preference.

Human studies support the involvement of CRF/CRF_1_ in alcohol dependence (23, 66), despite the fact that clinical trials for CRF_1_ antagonists have been unsuccessful to date (23). Elevated CRF levels were seen in the cerebrospinal fluid of recently withdrawn alcoholics (67). Similar to the *Crhr1* promoter variants seen in the msP line, human *CRHR1* SNPs have been associated with facets of alcohol dependence. Variant *CRHR1* haplotypes in adolescents predicted future problem drinking patterns, including binge drinking and lifetime prevalence of intoxication and alcohol dependence (68). *CRHR1* SNPs also predicted higher levels of alcohol consumption in already dependent individuals (68) and associated with reduced P300 levels, which are observed in alcoholics (69). *CRHR1* SNPs predicted greater future drinking in relation to recent stress history (70), implicating CRF_1_ systems in stress-induced drinking, consistent with observations in msP rats (31, 35), C57BL/6J mice (12) and *Crhr1* knockout mice (71). Genetic associations of CRF signaling to alcohol phenotypes in humans is not restricted to *CRHR1*, because several studies link genetic variants in *CRHBP*, which encodes the CRF-binding protein, to features of alcohol dependence. Polymorphisms in *CRHBP*, which is hypothesized to modulate the amount of CRF or urocortin-1 available to interact with its receptors, have been related to decreased EEG alpha wave power (72), a feature observed in alcoholics (72, 73), and are more prevalent in alcoholics with comorbid anxiety disorders (74). *CRHBP* polymorphisms have also been related to severity of stress-induced alcohol craving (75) and have been hypothesized to impact anxiety or drinking in alcohol-dependent individuals (76). Individual *CRHBP* and *CRHR1* SNPs have been shown to coordinately predict alcohol use disorder comorbidity in a panel of schizophrenic patients (77). Importantly, Ribbe et al. (77) demonstrated elevated levels of *CRHR1* mRNA relative to *CRHBP* mRNA in blood from individuals carrying the dual polymorphism, suggesting that CRF_1_ activation by CRF or urocortin-1 predominates over CRF/CRF-BP or urocortin-1/CRF-BP interactions in individuals with high predisposition to develop alcohol use disorders. Additional gene variants of *CRHR1* and *CRHBP* have been associated with various mood and anxiety disorders as well as alcohol use disorders [reviewed in Ref. (23)], implicating the CRF_1_ system in various mental illnesses characterized by emotional dysregulation. These human findings resonate with the present data in suggesting a key role for CRF_1_ signaling in some forms of alcoholism, perhaps particularly those with stress history or comorbid anxiety.

### *Crhr1* Promoter Genotype and Caloric Intake

Expression of the dual polymorphism in the *Crhr1* promoter was associated with increased alcohol preference, although preference levels were near ceiling in all genotypes and differences appear to result from decreased water intake rather than significantly increased alcohol intake *per se*. However, msP AA rats also consumed significantly less total calories than rats of the GA genotype, and tended to consume less food than both GG and GA genotype rats. While these results initially suggested a possible shift in calorie focus toward alcohol for AA msP rats—as observed in human alcoholics who transition toward consuming half their daily calories in alcohol (78, 79)—direct assessment of this possibility showed no significant genotype effects on percent of calories obtained from alcohol. Instead, these results suggest that *Crhr1* promoter genotype plays a more general role in regulating caloric intake that may not be specific to alcohol. Both CRF_1_ agonist (80) and antagonist (17) treatments reduced food intake, as did genetic deletion of *Crhr1* (17), further supporting a general role for CRF_1_ in regulating intake nonspecifically to alcohol. Interestingly, deletion of urocortin-1 only reduced escalated alcohol intake, but not food or water intake (45), demonstrating the capacity for the CRF system to specifically regulate dysregulated alcohol consumption in the absence of global intake modulation. The lack of differential caloric intake in rederived msP rats in the absence of alcohol, as well as the fact that caloric intake only differed significantly between AA and GA msP rats, suggests that *Crhr1* promoter genotype does not globally modulate consumption but rather may impact intake in a more directed fashion, whether in the presence of alcohol or under conditions of elevated stress that are proposed to increase *Crhr1* expression. Determining the relationship between CRF_1_ system activity, stress, and alcohol consumption, particularly a possible shift toward the use of alcohol as an alternative calorie source, in the msP rat line remain important future lines of investigation.

## Conclusion

Here, we show that two joint variants in the distal promoter region of *Crhr1* are restricted to the msP line, relative to other alcohol-preferring strains, and associate with increased alcohol preference, although all rats display extremely high preference, as well as decreased water intake and reduced total caloric consumption. In light of the growing body of literature demonstrating associations between polymorphisms in human *CRHR1* and *CRFBP* and comorbid stress/anxiety and AUD, the present data support a continued focus on CRF_1_ as a therapeutic target not for the global AUD population, but rather for a subset of AUD individuals, particularly those with stress-related comorbidities. A pharmacogenomic approach that accounts for CRF system polymorphisms may have particular utility for the treatment of alcohol use disorders.

## Ethics Statement

These studies were carried out in accordance with the recommendations of the European Community Council Directive for the Care and Use of Laboratory Animals and the University of Camerino Internal Ethical Committee for Laboratory Animal Protection and Use (CEAPA), or in accordance with the NIH Guide for the Care and Use of Laboratory Animals and The Scripps Research Institute Institutional Animal Care and Use Committee (IACUC). The protocols were approved by the University of Camerino CEAPA and by the Scripps Research Institute IACUC.

## Author Contributions

ML analyzed data and wrote the manuscript; JW designed experiments, collected and analyzed data, and edited the manuscript; LA collected data and edited the manuscript; VS designed experiments, collected data, and edited the manuscript; RC designed experiments and edited the manuscript; GK designed experiments and edited the manuscript; EZ designed experiments and wrote the manuscript.

## Conflict of Interest Statement

GK and EZ are inventors on a patent for the composition and use of non-peptide CRF_1_ receptor antagonists (US20100249138). All other authors declare that the research was conducted in the absence of any commercial or financial relationships that could be construed as a potential conflict of interest. The reviewer DF and handling editor declared their shared affiliation.
